# Plasminogen activator inhibitor-1 as a potential marker for the malignancy of colorectal cancer

**DOI:** 10.1038/sj.bjc.6602743

**Published:** 2005-08-09

**Authors:** T Sakakibara, K Hibi, M Koike, M Fujiwara, Y Kodera, K Ito, A Nakao

**Affiliations:** 1 Gastroenterological Surgery, Nagoya University Graduate School of Medicine, Nagoya 466-8560, Japan

**Keywords:** plasminogen activator inhibitor-1, colorectal cancer, quantitative RT–PCR

## Abstract

To test the hypothesis that plasminogen activator inhibitor-1 (PAI-1) may serve as a candidate marker for the malignancy of colorectal cancer (CRC), we performed a quantitative RT–PCR for PAI-1 gene and evaluated the possible relationship between PAI-1 gene expression levels and clinicopathological findings in CRC. A significant increase in PAI-1 expression scores was observed in lymph node metastasis-positive CRCs (2.19±0.43) compared to negative ones (0.35±0.42) (*P*=0.0037) as well as in distant metastasis-positive CRCs (3.50±1.18) compared to negative ones (0.99±0.30). The PAI-1 expression score markedly increased with the tumour stage (*P*=0.0063; ANOVA test). Moreover, multivariate analysis revealed the PAI-1 expression score to be a strong and independent prognostic factor for CRC (*P*=0.0432). These results suggested that PAI-1 might serve as a new parameter for the prediction of prognoses in CRC.

There is now good evidence that a series of genetic alterations in both dominant oncogenes and tumour suppressor genes are involved in the pathogenesis of human colorectal cancer (CRC). Activation of oncogenes such as the *ras* gene, and inactivation of tumour suppressor genes such as the *APC* and *p53* genes, have been identified in CRC (Bos *et al*, 1987; Baker *et al*, 1990; Nishisho *et al*, 1991). In addition, we found that several other genes are related to the pathogenesis of this disease (Hibi *et al*, 1996, 1997, 2002, 2004; Yamazaki *et al*, 2002). An investigation of genetic changes is important to clarify the tumorigenic pathway of CRC (Vogelstein *et al*, 1988).

Plasminogen activator inhibitor-1 (PAI-1), a 45-kDa serine proteinase inhibitor with a reactive peptide bond, Arg345–Met346, is a multifaceted proteolytic inhibitor that not only functions as a fibrinolytic inhibitor, but also plays an important role in signal transduction, cell adherence, and cell migration. There is clinical evidence implicating PAI-1 as a key factor in tumour invasion and metastasis (Potempa *et al*, 1994; Lijnen, 2005). Moreover, PAI-1 has been linked to a poor prognosis in several cancers (Nekarda *et al*, 1994; Cantero *et al*, 1997; Chambers *et al*, 1998; Foekens *et al*, 2000; Konecny *et al*, 2001). Previously, we also demonstrated that PAI-1 expression was significantly correlated with a poor prognosis in esophageal squamous carcinoma (Sakakibara *et al*, 2004). These results prompted us to examine the PAI-1 expression level in other cancers, especially in CRC.

To test the hypothesis that PAI-1 may serve as a candidate marker for the malignancy of CRC, we performed a quantitative reverse transcription-PCR (RT–PCR) and evaluated the relationship between the PAI-1 gene expression levels and clinicopathological findings in CRC.

## MATERIALS AND METHODS

### Patients and tissue specimens

The study group consisted of 55 CRC patients (mean age 64.5 years; range 41–85 years), who underwent surgical operations at the Gastroenterological Surgery of the Nagoya University Graduate School of Medicine from 1994 to 2002. Written informed consent, as indicated by the institutional review board, was obtained from all patients. All patients were followed at our hospital with periodical examinations. The median follow-up was 33 months. In total, 41, 4, and 10 patients received R0, R1, and R2 resection, respectively. Although no patients received neoadjuvant therapy, 25 patients received adjuvant therapy. The distribution of these patients was not related to PAI-1 expression scores. A total of 17 patients have been lost to follow-up until now. All tumours and corresponding normal tissues were collected at the surgical resection and stored at −80°C. They were graded according to their tumour-node-metastasis (TNM) stage as follows: 12 had stage I disease; 12 had stage II; 23 had stage III; and eight had stage IV. The patients were classified into two groups according to age, sex, histology, tumour size, tumour site, depth of tumour invasion, lymph node metastasis, perineal dissemination, carcinoembryonic antigen (CEA), and TNM stage.

### RNA preparation and reverse transcription

Total RNA was extracted from CRC and corresponding normal tissues with guanidium thiocyanate as described previously (Hibi *et al*, 1996). The amount of RNA was measured spectrophotometrically by absorbance at 260 nm. First-strand cDNA was generated from RNA as described previously (Hibi *et al*, 1991).

### Quantitative RT–PCR

Quantitative RT–PCR was performed in an ABI sequence detection system 7000 using SYBR Green PCR Master Mix (Applied Biosystems, Foster City, CA, USA). Thermocycling was carried out at a final volume of 50 *μ*l containing 2.0 *μ*l of the cDNA sample, 1.0 *μ*l each of the PAI-1 primers (forward and reverse), and 25 *μ*l of Mix SYBR Green I/Enzyme (including Taq DNA polymerase, reaction buffer, and deoxynucleotide triphosphate mixture). The PAI-1 primers for quantitative PCR were described previously (Castello *et al*, 2002). PCR amplification consisted of 50 cycles (95°C for 15 s, 60°C for 60 s, and 72°C for 18 s) after an initial denaturation step (95°C for 10 min). To correct for differences in both quality and quantity among samples, GAPDH was used as an internal control. GAPDH primers were purchased from Applied Biosystems. Plasminogen activator inhibitor-1 and GAPDH mRNA variability were determined from triplicate samples, the quantity of which was in error by less than 10%. We applied an average quantity of triplicated samples. The targets were obtained from the same mRNA preparations.

### PAI-1 expression score

We calculated the relative amounts of CRC (T) and corresponding normal tissue (N) mRNA that we had normalised to an internal control GAPDH mRNA. Next, we applied the logarithmic scale as described previously (Pasco *et al*, 2001; Wang *et al*, 2003; Sakakibara *et al*, 2004). We then defined the PAI-1 expression score as follows: 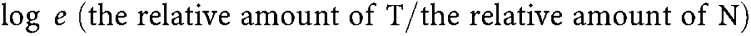


### Statistical and multivariate analyses

Data were expressed as means±s.e. Differences between the means of analysed variables observed were calculated by the Student’s *t*-test. The significance in correlations between tumour stages and variables was determined by one-way ANOVA. *P*<0.05 (two-tailed) was considered significant. Survival rates were calculated by the Kaplan–Meier method for analysis of censored data. To compare the prognostic significance of the PAI-1 expression score with that of other parameters, multivariate analysis was performed. Variables were eliminated from the model one by one in a backward fashion and re-included only if the *P*-value was less than 0.05.

## RESULTS

We analysed PAI-1 expression levels in 55 CRC samples using a quantitative RT–PCR. The mRNA concentrations were determined after an extensive optimisation of PCR conditions, including MgCl_2_ concentrations, reaction temperature, and cycling times. This provided us with a highly sensitive, specific, and reproducible real-time RT–PCR for specific detection of these mRNAs.

**Figure 1:**
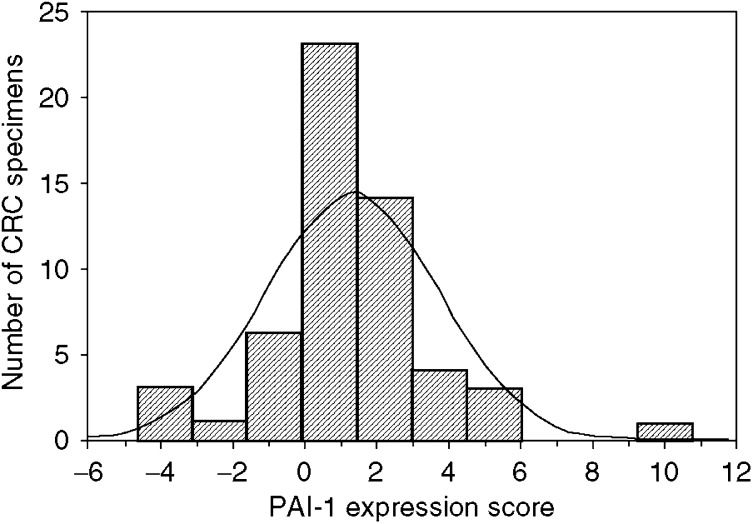
Distribution of PAI-1 expression scores in CRC (average was 1.35±0.32).

**Figure 2:**
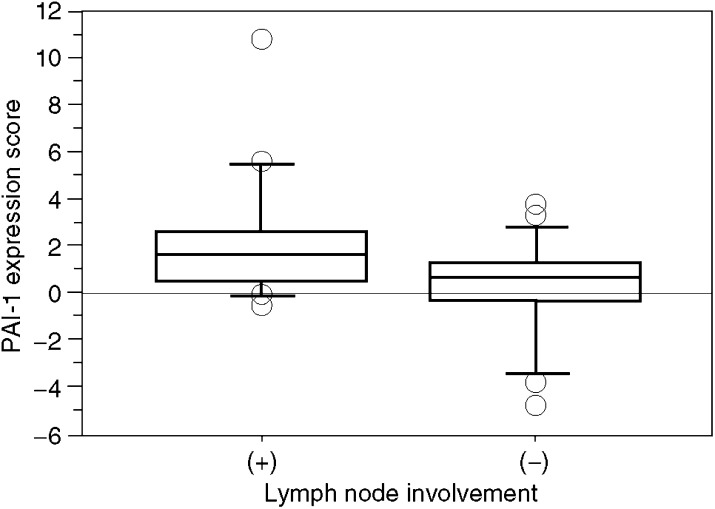
Difference in PAI-1 expression scores according to lymph node metastasis. Upper and lower limits of boxes, and line across boxes, indicate the 75th and 25th percentiles, and the median, respectively. Upper and lower horizontal bars indicate maximal and minimal scores, respectively. Outliers are illustrated as circles. Plasminogen activator inhibitor-1 expression scores were 2.19±0.43 in metastasis-positive CRCs and 0.35±0.42 in metastasis-negative ones (*P*=0.0037; Student's *t*-test).

**Figure 3:**
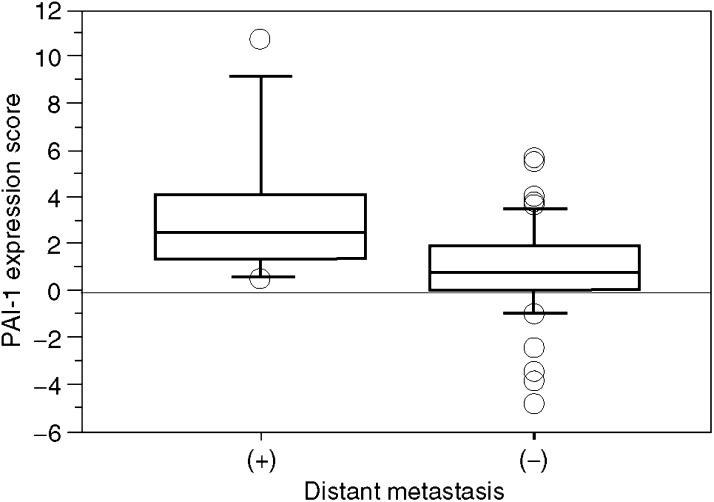
Difference in PAI-1 expression scores according to distant metastasis: 3.50±1.18 in metastasis-positive CRCs, and 0.99±0.30 in metastasis-negative ones (*P*=0.0052; Student's *t*-test).

**Figure 4:**
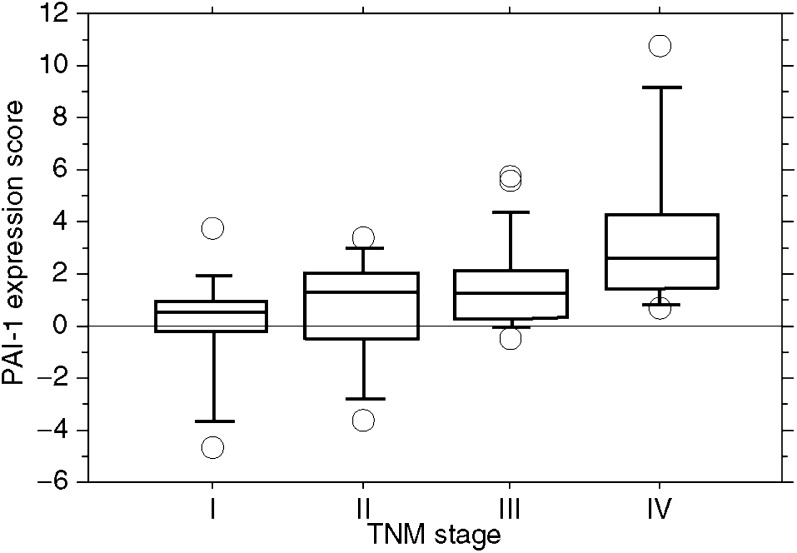
Differences in PAI-1 expression scores according to tumour stages: 0.01±0.63 in stage I, 0.66±0.61 in stage II, 1.67±0.0.36 in stage III, and 3.50±1.18 in stage IV (*P*=0.0063; ANOVA).

**Figure 5:**
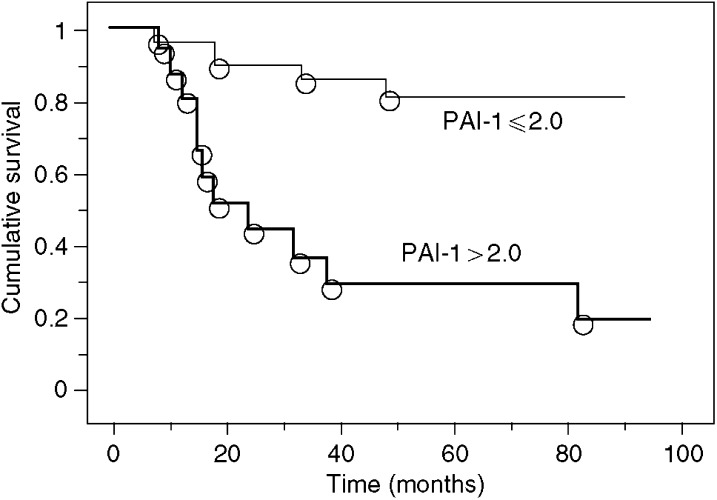
Cumulative survival of patient groups according to PAI-1 expression scores (more or less than 2). High PAI-1 expression-score group showed significantly poorer survival rates than the low PAI-1 expression-score group (*P*<0.0001).

To determine the role of PAI-1 expression in CRC, we examined the correlation of CRC expression scores with the clinicopathological features. Figure 1 shows the distribution of the PAI-1 expression scores (the average was 1.35±0.33). Figure 2 shows the differences in PAI-1 expression scores according to lymph node metastasis. A significant increase in PAI-1 expression scores was observed in lymph node metastasis-positive CRCs (2.19±0.43) compared to negative ones (0.35±0.42) (*P*=0.0037). Figure 3 shows the differences in PAI-1 expression scores according to distant metastasis. A significant increase in PAI-1 expression scores was observed in distant metastasis-positive CRCs (3.50±1.18) compared to negative ones (0.99±0.30). These results are summarised in Table 1. As shown in Figure 4, the PAI-1 expression score was significantly increased with the tumour stage (stage I=0.01±0.63, stage II=0.66±0.61, stage III=1.67±0.36, stage IV=3.50±1.18) (*P*=0.0063; ANOVA test). We then examined the cumulative survival of patient groups according to their PAI-1 expression scores (more or less than 2). Interestingly, the high PAI-1 expression-score group showed significantly poorer survival rates than the low PAI-1 expression-score group (Figure 5, *P*<0.0001).

To confirm the prognostic significance of the PAI-1 expression score, other clinicopathological variables that might affect survival were further analysed by Cox regression analysis. In univariate analysis, the depth of tumour invasion (*P*=0.0154), lymph node involvement (*P*<0.0001), distant metastasis (*P*<0.0001), and PAI-1 expression score (*P*<0.0001) were significantly correlated with survival (Table 2). To determine the independent value and the relative risk (RR) of these prognostic factors, multivariate analysis was performed. Two prognostic factors were found to be independent values: lymph node metastasis (*P*=0.0267) and PAI-1 expression score (*P*=0.0432). Taken together, these findings showed that the PAI-1 expression score constituted a strong and independent prognostic factor for CRC.

## DISCUSSION

The plasminogen activation system plays a role in cancer progression, presumably via extracellular matrix degradation and tumour migration (Pedersen *et al*, 1994). It is generally believed that serine protease, a urokinase-type plasminogen activator (uPA), initiates a proteinase cascade at the cell surface and promotes tumour invasion and angiogenesis. Urokinase-type plasminogen activator is frequently overexpressed in several cancers and is a strong prognostic indicator for decreased patient survival rates (Umeda *et al*, 1997; Duffy *et al*, 1999). Plasminogen activator inhibitor-1, the protease inhibitor, is mainly synthesised in vascular endothelial cells and regulates fibrinolytic activity in the vasculature by controlling uPA activity. Recently, the involvement of PAI-1 in tumour growth was suggested because of its high expression levels in tumour extracts. At first, PAI-1 was expected to inhibit tumour progression by inhibiting uPA activity on the tumour cell surface. However, prognostic studies have indicated that PAI-1 is also a clinical marker for a poor prognosis in a variety of human cancers, suggesting that it plays an important role in promoting tumour progression and invasion (Grondahl-Hansen *et al*, 1993; Cho *et al*, 1997; Knoop *et al*, 1998). No clear explanation has yet been found for this apparent paradox. Although the exact tumour biological functions of PAI-1 remain uncertain, it is expressed in multiple cell types and has multiple molecular interactions. This discrepancy could be due to a difference in tumour histology, or it may merely reflect the biological tumour features of different types of cancer.

Tumour growth and metastasis are angiogenesis-dependent. A tumour must continuously stimulate the growth of new capillary blood vessels to promote its growth. Furthermore, angiogenesis is required for tumour cells to enter the circulation and metastasise to distant sites, such as liver, lung, or bone. Tumour cells simultaneously secrete proteases (uPA) and their inhibitors (PAI-1), and the balance between the two precisely regulates the level of extracellular proteolysis, thus either promoting or suppressing angiogenesis (Folkman *et al*, 2001). It is likely that excess PAI-1 decreases cell adhesion to the extracellular matrix by interfering with uPAR binding to vitronectin, thus facilitating cell invasion and migration (Abe *et al*, 1999). Other reports have also indicated that excess PAI-1 plays an important role in tumour growth and metastasis by stimulating angiogenesis (McMahon *et al*, 2001; Bajou, 2002; Devy *et al*, 2002). Recently, it has been reported that deficient PAI-1 expression in host mice prevented local invasion and tumour vascularisation (Bajou *et al*, 1998). When this PAI-1 deficiency was circumvented by an intravenous injection of a replication-defective adenoviral vector expressing human PAI-1, the invasion and its associated angiogenesis resumed. This experimental evidence demonstrated that host-produced PAI-1 is essential for cancer cell invasion and angiogenesis (Gutierrez *et al*, 2000; Bajou *et al*, 2004).

In this study, we demonstrated for the first time that PAI-1 expression increased with the CRC stage and was associated with a poor prognosis. In univariate analysis, the PAI-1 expression score was significantly correlated with survival of CRC. Moreover, in multivariate analysis, the PAI-1 expression score was a strong and independent prognostic factor in CRC, second to the lymph node involvement. These results suggested that PAI-1 might serve as a new parameter for the prognostic prediction of CRC.

## Figures and Tables

**Table 1:** Clinicopathological features and PAI-1 expression scores in colorectal cancer

**Clinicopathological feature**	**Variable**	**No. of cases score**	**PAI-1 expression**	***P* value^a^**
Age	<70	37	1.48±0.42	0.59
	⩾70	18	1.10±0.51	
				
Sex	Male	37	1.63±0.41	0.27
	Female	18	0.87±0.52	
				
Histology	tub^b^	43	1.25±0.40	0.54
	por, muc, sig^c^	12	1.74±0.46	
				
Tumour size (mm)	<50	31	1.49±0.71	0.45
	⩾50	24	0.92±0.35	
				
Tumour site	C, A, T^d^	14	1.17±0.47	0.75
	D, S, R^e^	41	1.41±0.41	
				
Depth of tumour invasion	T1–T2	38	0.84±0.30	0.016
	T3–T4	17	2.51±0.75	
				
Lymph node metastasis	N0	25	0.35±0.42	0.0037
	N1–N2	30	2.19±0.43	
				
Distant metastasis	−	47	0.99±0.30	0.0052
	+	8	3.50±1.18	
				
Peritoneal dissemination	−	51	1.23±0.34	0.17
	+	4	2.97±0.96	

a Student's *t*-test.

b tub=tubular adenocarcinoma.

c por=poorly differentiated adenocarcinoma; muc=mucinous adenocarcinoma; sig=signet-cell adenocarcinoma.

d C=cecum; A=ascending colon; T=transverse colon.

e D=descending colon; S=sigmoid colon; R=rectum.

**Table 2:** Risk factors for overall survival rate determined by univariate or multivariate analysis in colorectal cancer

**Clinicopathological feature**	**Variable**	**Univariate**	**Multivariate**	**RR^a^**	**95% CI^b^**
Lymph node	N0/N1–N2	<0.0001	0.0267	11.0	1.3–92
Depth of tumour invasion	T1–T2/T3–T4	0.0094	0.9850	1.0	0.23–4.3
Distant metastasis	±	<0.0001	0.2759	2.5	0.47–13
Peritoneal dissemination	±	0.0009	0.5978	1.5	0.32–7.0
PAI-1	⩽2.0/2.0<	<0.0001	0.0432	3.1	1.0–9.6

a Relative risk.

b Confidence interval.
